# Assessment of a Protocol for Reducing Indwelling Urinary Catheter Usage: Reduced Infection Without Increased Acute Kidney Injury

**DOI:** 10.7759/cureus.63578

**Published:** 2024-07-01

**Authors:** Stephen Roche, Raymond Okeke, John Culhane

**Affiliations:** 1 Surgery, Saint Louis University, Saint Louis, USA

**Keywords:** creatinine kinase, trauma, acute kidney injury, urinary tract infection, indwelling urinary catheter

## Abstract

Introduction

In 2019, a level one trauma center in St. Louis, Missouri launched a campaign to reduce the use of indwelling urinary catheters (IUC) in the trauma population. Our study assesses whether the campaign achieved the intended effect of reducing catheter-associated urinary tract infection (CAUTI) and whether this came at the cost of increased acute kidney injury (AKI).

Methods

We examined a cohort of patients from before and after the IUC reduction campaign. We compared days with IUC, UTI, CAUTI, and AKI, both recorded in the registry and based on a direct review of laboratory results. Significance testing is performed with the Student’s t-test for continuous variables and Fisher’s exact test for categorical variables. For multivariate analysis, multivariate linear regression is used for continuous outcomes, and multivariate logistic regression is used for binary categorical outcomes.

Results

On average, the post-campaign cohort was older and more severely injured. There was a modest decrease in IUC usage following the campaign, which was significant when adjusted for common trauma covariates, B=-0.93; p=0.04. There were 10 (0.4%) cases of CAUTI in the pre-intervention group versus 0 post-intervention (p=0.002). Pyuria was significantly lower post-intervention: 118 (40.3%) versus 84 (29.3%), p=0.007. This remained significant on multivariate analysis: odds ratio (OR): 0.52, p=0.008. There were no significant differences in creatinine (Cr) absolute values or temporal trends over the course of admission between the cohorts. There was no increased AKI measured by kidney disease improving global outcomes (KDIGO) criteria.

Conclusion

The IUC reduction protocol was associated with a significant decrease in CAUTI without a significant increase in AKI.

## Introduction

The use of indwelling urinary catheters (IUC) is common in traumatically injured patients. These catheters assist immobile patients in voiding, simplify hygiene, and allow for precise measurement of urinary output, which can be used as an index of resuscitation and fluid balance. While useful for patient care, IUC may result in undesired complications. Prominent among these is catheter-associated urinary tract infection (CAUTI), which has come under increased scrutiny as a preventable complication subject to financial penalty [1].

As a result, hospitals have enacted measures to reduce CAUTI. Since the main risk factor for CAUTI is the catheter itself, a key strategy is to limit the use of IUC to appropriate indications. Implementation of protocols to reduce IUC along with other strategies has resulted in decreased incidence of CAUTI in hospitals throughout the United States [2-4]. While reducing CAUTI is a worthy goal, quality improvement protocols may have unintended consequences. Traditionally IUCs have been considered an essential tool for resuscitation of critically ill patients, including patients with severe trauma. Urine output is a sensitive measure of renal perfusion and hourly fluctuation can alert a provider to adjust fluid intake, which may reduce the risk of acute kidney injury (AKI) [5].

In 2019, a level one trauma center in St. Louis, Missouri launched a campaign to reduce the use of IUC in the trauma population. This provides the setting for a natural experiment: to examine the outcomes of traumatically injured patients before and after the policy implementation. Our hypothesis is that the campaign to limit IUCs is associated with reduced CAUTI at the cost of increased AKI.

## Materials and methods

In 2019, an urban level one trauma hospital instituted a policy intended to reduce CAUTI. The policy included measures related to the insertion and maintenance of IUC, but the focus was on reducing their use as much as possible. The protocol included reviewing a checklist on rounds, creation of order sets in the electronic medical record to discourage routine or unnecessary IUC, reminders to providers to remove unnecessary IUC, nursing empowerment to remove IUC that met certain criteria without an explicit order, and close monitoring of CAUTI with discussion at scheduled meetings.

The design is a retrospective historical cohort study. We examined a cohort of patients before and after the protocol implementation. First, we examined a data set of all patients in the trauma registry admitted during 2017 and 2021 looking at AKI, UTI, and CAUTI incidence. To analyze IUC usage and complications in more detail, we then performed a direct chart review of 300 severely injured patients from 2017 compared with the same number from 2021. The final analysis contained slightly fewer patients due to incomplete records and exclusions. Inclusion criteria were injury severity score (ISS) >15 and length of stay (LOS) >2 days.

Analysis was restricted to the index admission. Demographic and injury characteristic variables were collected for baseline comparison. Chart review was used to determine the presence of an IUC for every day of admission. We excluded IUCs kept in place due to urogenital injury because these were not subject to the protocol. Derived values were calculated. The sum of IUC days is the total days during the admission in which an IUC was present for any length of time. IUC day percent is the sum of IUC days divided by LOS in days times 100. IUC free days is the LOS minus the sum of IUC days. 

Every creatinine (Cr) value during the admission was recorded. For cases with more than one Cr value per day, we calculated the sum, average, and maximum of the values. These values were then used to create summary statistics for each patient. Delta Cr is the difference between the initial and maximum Cr. We identified the patients who met kidney disease improving global outcomes (KDIGO) criteria for acute renal failure. These consist of a Cr increase ≥0.3 within the first 48 hours or a Cr increase of 1.5 times baseline within the first seven days.

We summarized the urinalysis (UA) data by organizing it into results consistent with bacteriuria, pyuria, or hematuria at any time during the admission. Bacteriuria was defined as any value above the upper reference for bacteria (more than “Trace”). Pyuria was defined as any value above the upper reference for white blood cells (WBC), leukocyte esterase, or nitrite. Hematuria was defined as any value above the upper reference for red blood cells (RBC) or blood.

For univariate analysis, we used Student’s t-test for continuous variables and Fisher’s exact test for categorical variables. For multivariate analysis, we used multivariate linear regression for continuous outcome and multivariate logistic regression for binary categorical outcome. The predictor variable of interest for both is status-post-IUC campaign. Covariates for both are age, gender, ISS, trauma score and injury severity score (TRISS), body mass index (BMI), LOS, and blunt versus penetrating mechanism.

The statistical tools used are the Python SciPy library, ROOT, and the R project. The significance level is set at p <0.05.

## Results

Table 1 describes demographics and baseline characteristics. Common demographic and injury severity parameters are presented. Data is presented as mean (±SD) for continuous variables and n (%) for categorical variables. The 2021 cohort is slightly older with more female patients. There is a higher percentage of blunt trauma for 2021. The other characteristics are closely matched.

**Table 1:** Displays demographics and injury characteristics. SD, standard deviation; SMD, standardized mean difference; ISS, injury severity score; TRISS, trauma score and injury severity score; BMI, body mass index; LOS, length of stay

Patient/injury characteristic	2017	2021	SMD
n	293	287	
Age (mean (SD))	46.21 (±22.07)	50.03 (±21.86)	0.175
Gender n (%)			0.135
Female	83 (28.3)	95 (33.1)	
Male	210 (71.7)	191 (66.6)	
Injury type n (%)			0.153
Blunt	237 (80.9)	245 (85.4)	
Penetrating	56 (19.1)	41 (14.3)	
ISS (mean±SD)	24.90 (±9.03)	24.41 (±9.71)	0.052
TRISS (mean±SD)	0.86 (±0.22)	0.84 (±0.23)	0.108
BMI (mean±SD)	27.26 (±6.39)	27.56 (±6.86)	0.045
LOS (days) (mean±SD)	13.22 (±10.40)	14.07 (±12.44)	0.074

The 2021 cohort is slightly older with more female patients. There is a higher percentage of blunt trauma for 2021. The other characteristics are closely matched.

Table 2 summarizes IUC usage for the cohorts selected for detailed direct chart review and the prevalence of indwelling catheters for cohorts from before the IUC initiative (2017) versus after the IUC initiative (2021). Data is presented as mean (±SD) for continuous variables and n (%) for categorical variables.

**Table 2:** IUC usage for the cohorts selected for detailed direct chart review. IUC, indwelling urinary catheter

IUC exposure	2017	2021	SMD
n	293	287	
Ever had IUC = Yes (%)	167 (57.0)	170 (59.2)	0.045
Average IUC days (mean±SD)	4.77 (±7.72)	4.35 (±7.033)	0.056
Percent days with IUC (mean±SD)	28.33 (±31.82)	25.70 (±30.61)	0.084
IUC free days (mean±SD)	8.44 (±6.96)	9.72 (±9.41)	0.154

Multivariate analysis shows that the cohort following the IUC campaign had an adjusted decrease of 0.93 IUC days versus the cohort prior to the intervention: predictor: post-Foley campaign; estimate: -0.93 standard error 0.45 t-value -2.10, p=0.04.

Table 3 displays selected complications for all trauma patients admitted for the years 2017 and 2021. These are complications potentially related to IUC pre- and post-IUC initiatives. The population is all trauma patients admitted for the years 2017 and 2021. Data is presented as n (%).

**Table 3:** Selected complications for all trauma patients admitted for the years 2017 and 2021. UTI, urinary tract infection; CAUTI, catheter-associated urinary tract infection; AKI, acute kidney injury

Complication	2017	2021	P
N	2502	2959	
UTI yes (%)	11 (0.4)	9 (0.3)	0.548
CAUTI yes (%)	10 (0.4)	0 (0.0)	0.002
AKI yes (%)	9 (0.4)	13 (0.4)	0.804

The following results refer to the cohorts we extracted for detailed direct chart review. Table 4 displays the univariate analysis of UTI-related laboratory values for patients selected from the years 2017 and 2021. Data is presented as n (%).

**Table 4:** UA results from the cohorts pre- and post-IUC initiative. UA, urinalysis; UTI, urinary tract infection; IUC, indwelling urinary catheter

UTI laboratory evidence	2017	2021	p
n	293	287	
Pyuria yes (%)	118 (40.3)	84 (29.3)	0.007
Bacteriuria yes (%)	81 (27.6)	100 (34.8)	0.075
Hematuria yes (%)	145 (49.5)	128 (44.6)	0.273

Table 5 displays the result of multivariate logistic regressions with the outcome of UTI-related laboratory values and the predictor of status post-IUC campaign adjusted for the covariates specified in the methods section. The odds of a patient testing positive for pyuria on UA after the IUC campaign is 0.52 versus before the IUC campaign.

**Table 5:** Adjusted OR of UTI-related laboratory values before and after the IUC campaign. OR, odds ratio; SE, standard error; UTI, urinary tract infection; IUC, indwelling urinary catheter

UTI laboratory evidence	OR	Coefficient	SE	Wald Z	P
Outcome: pyuria					
Post-IUC campaign	0.52	-0.6481	0.1934	-3.35	0.0008
Outcome: bacteriuria					
Post-IUC campaign	1.29	0.2525	0.1893	1.33	0.1823
Outcome: hematuria					
Post-IUC campaign	0.75	-0.2836	0.1827	-1.55	0.1207

Table 6 displays the Cr changes before and after the Foley campaign and patients in each cohort who met KDIGO criteria for AKI. The n for KDIGO comparison does not match previous tables because some patients did not have enough days of Cr measured to calculate KDIGO criteria. Data is presented as mean (±SD) for continuous variables and n (%) for categorical variables.

**Table 6:** Univariate analysis of changes in Cr before and after the IUC initiative. Cr, creatinine; IUC, indwelling urinary catheter; AKI, acute kidney injury; KDIGO, kidney disease improving global outcomes

Cr	2017	2021	p
n	293	287	
Initial Cr (mean±SD)	1.02 (±0.32)	1.09 (±0.94)	0.250
Average Cr (mean±SD)	0.84 (±0.30)	0.89 (±0.61)	0.207
Max Cr (mean±SD)	1.14 (±0.42)	1.24 (±1.07)	0.183
Delta Cr (mean±SD)	0.12 (±0.26)	0.15 (±0.37)	0.393
n	267	287	
KDIGO AKI yes (%)	19 (7.1)	23 (8.0)	0.690

Multivariate analysis with linear regression shows no significant change in average Cr following the IUC campaign. The slope of the regression is 0.053. The standard error is 0.042; p=0.21.

## Discussion

Our trauma registry did not show an increase in AKI following the Foley campaign (Table 3) but as with CAUTI, AKI may also be underreported in large registries [6]. To address this shortcoming, we directly examined the laboratory data for evidence of AKI. KDIGO guidelines have provided a standardized definition and staging system for AKI [7]. These criteria are designed to be sensitive, reflecting the idea that even a small and reversible decrease in renal function may be clinically important. We chose to use this sensitive measure to assess whether our IUC policy was associated with any compromise of renal function. 

To be thorough, we examined Cr in several ways including average, maximum, maximum increase during admission, and AKI defined by KDIGO criteria (Table 6). Neither univariate nor multivariate analysis showed an increase in renal failure associated with the IUC campaign. Thus, we conclude that the policy is safe with respect to fluid resuscitation and maintenance.

A major goal of trauma care is to prevent secondary damage during the healing process [8]. This includes avoiding complications such as hospital-acquired infection and AKI. IUCs are commonly used during inpatient care and may be agents of both benefit and harm. The benefits of IUCs include accurate recording of fluid intake and output, facilitated hygiene, relief of mechanical or functional urinary outlet obstruction, and treatment of anatomic disruption such as urethral injury. Complications include traumatic insertion, traumatic self-extraction, erosion of genitourinary structures, and CAUTI [1]. The last complication, CAUTI, has come under increasing scrutiny in recent years. 

In 2014, the Affordable Care Act required Medicare to begin reducing payments to hospitals in the lowest-performing quartile with respect to certain hospital-acquired conditions such as CAUTI. These financial penalties placed tremendous pressure on hospitals to report fewer infections [9]. To reduce CAUTI, many hospitals have implemented nursing-driven protocols to decrease IUC use. Studies of these protocols have shown lower rates of days with an IUC and lower CAUTI rates [10-12]. Our results are consistent with these reports. For the two periods we examined, we went from 10 CAUTIs in the earlier year to complete eradication in the latter.

We achieved these results with a reduction in Foley days that was measurable but modest (Table 2). One might ask how a small change in Foley usage could lead to such a dramatic reduction in CAUTI. One consideration is that there was some imbalance in baseline characteristics. LOS, ISS, and BMI were higher in the more recent cohort. Thus, the latter cohort was sicker with an expected higher use of invasive devices. To assess the effect of this imbalance, we ran a multivariate analysis correcting for common indices of trauma severity. This revealed a significant reduction in adjusted Foley days. Despite being sicker with an arguably stronger indication for an IUC, the later cohort of patients was spared indwelling catheter days.

Another possibility is a change in coding practice. The threat of financial penalties may influence the coding and reporting of complications. Coding is inherently subjective; direct chart review and clinical databases often report higher complication rates than those recorded in the hospital-acquired condition reduction program (HACRP) administrative database [13,14]. We addressed this possibility by examining the most objective available measure of UTI:UA (Tables 4, 5). Instead of relying on data abstraction and reporting, which is dependent on the judgment of coders and registrars, we looked directly at the laboratory data. Positive findings on UA are not pathognomonic of UTI but support the diagnosis in the right clinical setting. Less pyuria in the later cohort is objective evidence of a change in urine quality following the IUC campaign.

Another possibility is that components of a Foley bundle other than usage reduction may have contributed to the improvement in the CAUTI rate. Improved insertion and maintenance techniques are also important in avoiding CAUTI [2]. The premise of penalizing hospitals for certain hospital-acquired conditions is that they are surrogates for lower overall quality of care. It is possible that our lower CAUTI rate is a sign of improvement in overall patient safety.

The prospect of both economic penalty and damage to institutional reputation due to reportable hospital-acquired conditions may lead to unintended consequences. When clinicians are pressured to remove IUCs early to decrease CAUTI, they may be removed prematurely. This may lead to urinary retention and associated complications [15]. One purpose of an IUC is accurate measurement of fluid intake and output allowing providers to recognize oliguria early and treat it promptly. Oliguria may be a sign of decreased renal perfusion. Uncorrected hypoperfusion may result in parenchymal damage to the kidney leading to ATN and AKI. Renal failure in trauma is associated with an overall poor prognosis, including increased mortality [16].

More accurate and timely measurement of urine output made possible by an IUC may serve as a valuable index of resuscitation. Shalman et al. found that minute-by-minute urinary flow rate and urinary flow rate variation decreased with sepsis and increased with resuscitation. Thus, short-term change in UO was shown to be an early and sensitive signal of hypoperfusion [5].

Strengths

We assess the association of decreased Foley usage not only with CAUTI but also with renal failure, which is less frequently studied. We use direct chart review with objective measures of UA and Cr instead of relying only on registry data. We use the most sensitive measure of AKI from the KDIGO guidelines.

Limitations

This is a retrospective chart review. Associations may not reflect cause and effect. As with any historical control study, differences between groups may arise from factors other than the studied intervention. In addition, this is a single-center data analysis; results may not be generalizable to other hospitals or healthcare systems. Finally, as only adult patients were included in this analysis, results may not be generalizable to pediatric patients.

## Conclusions

Implementation of a protocol to reduce IUC usage at a level one trauma center was associated with a significant decrease in CAUTI reported in the trauma registry. Direct review of laboratory data supports the accuracy of the registry. IUC days were reduced but not by a large amount. Other parts of the bundle may be responsible for the benefit. There was no significant increase in the incidence of AKI, even when measured by the most sensitive criteria of the KDIGO classification. Thus, the IUC campaign was associated with diminished CAUTI without the unintended consequence of increasing AKI.
